# Fetal treatment of sacrococcygeal teratoma: state of the art

**DOI:** 10.3389/fped.2025.1410269

**Published:** 2025-06-10

**Authors:** M. C. Cianci, G. Fusi, F. Morini, E. Severi, A. Morabito, C. Grimaldi

**Affiliations:** ^1^ Department of Neurosciences, Psychology, Drug Research and Child Health (NEUROFARBA), University of Florence, Florence, Italy; ^2^ Department of Pediatric and Neonatal Surgery, Meyer Children's Hospital IRCCS, Florence, Italy

**Keywords:** fetus, sacrococcygeal teratoma, fetal treatments, *in utero* treatments, solid tumors

## Abstract

Antenatally detected sacrococcygeal teratoma (SCT) is a potentially life-threatening condition. Its prenatal management remains a topic of debate due to its association with fetal and maternal complications. This review delves into various fetal approaches to SCT, elucidating the roles of different procedures. Overall, fetal treatments are proposed to highly selected patients, with severe complications of SCT who carry a dismal prognosis and a high-risk *in utero* death. No shared protocols for patient and/or procedure selection exist thus overall management of these patients is highly dependent on the team skills and facility resources. Despite the general feeling that a prenatal diagnosis of SCT involves a high mortality risk, this comprehensive review demonstrates that advancements in fetal SCT treatment positively affect both fetal and maternal outcomes.

## Introduction

Sacrococcygeal teratoma (SCT) is one of the most common solid tumors in fetusus (1). Although mortality rate in neonatal SCT is quite low (survival of 83%–90%), the prognosis in prenatally diagnosed SCT tends to be poorer with mortality rates between 30% and 50%, therefore careful management and meticulous follow up are mandatory both in the short and long-term period (2–4). A specific field of investigation is therefore prenatal SCTs that show a significantly higher mortality, especially in case of hydrops development (1). Given its potentially life-threatening nature, fetal treatments emerge as a useful tool in selected cases (5–8). Over time, various strategies have been proposed based on fetus and mother's condition and specific tumor characteristics (9, 10).

Current indications for fetal treatments depend on the severity of clinical manifestations: the rapid growth, a predominant solid component and hypervascularization resulting in fetal hydrops, placentomegaly, polyhydramnios, and preterm labor (11–13). Maternal complications, such as the “mirror syndrome,” can also be indications to prenatal treatment (14, 15). Hence, fetal treatments are indicated for severe fetal SCT (FSCT) with the characteristics above, while FSCT without those complications are candidates for prenatal expectant management and subsequent neonatal surgery.

Despite notable progress, the management of fetal FSCT remains a topic of debate, complicated by the challenge of selecting the best treatment for each candidate (16, 17).

The aim of the current review is to provide a comprehensive view of the current landscape of in- utero approaches to severe FSCT. A comparison between outcomes of FSCT's patients submitted to fetal treatments and those with no fetal intervention is not discussed since indications are different. Limit of the current study is the absence of a statistically significant comparative analysis between techniques due to data heterogeneity and the amount of missing data.

## Materials and methods

PubMed and Embase were searched including studies from 1983 to September 2023 using the following keywords: (fetus OR fetal OR foetal) AND (prenatal OR intrauterin*) AND (sacrococcygeal teratoma).

Reference management was done with Rayyan. After removal of duplicates, three reviewers (G.C., M.C.C., G.F.) independently screened the titles and abstracts. The full texts of eligible articles were reviewed. Statistical analyses were performed using prisma graph-pad (R) and r. Data are presented as mean ± SD unless otherwise specified. Differences in categorical variables between two groups were analysed using Fisher's exact test and differences in continuous variables with unpaired t-tests. Statistical significance was set at *P* < 0.05. All tests were two-sided.

## Results

The literature search yielded 790 citations. After removal of 467 overlapping results, 323 abstracts and titles were screened. After screening, 48 manuscripts were retained for the final analysis [Supplementary Table 1 (18–65)].

**Figure 1:**
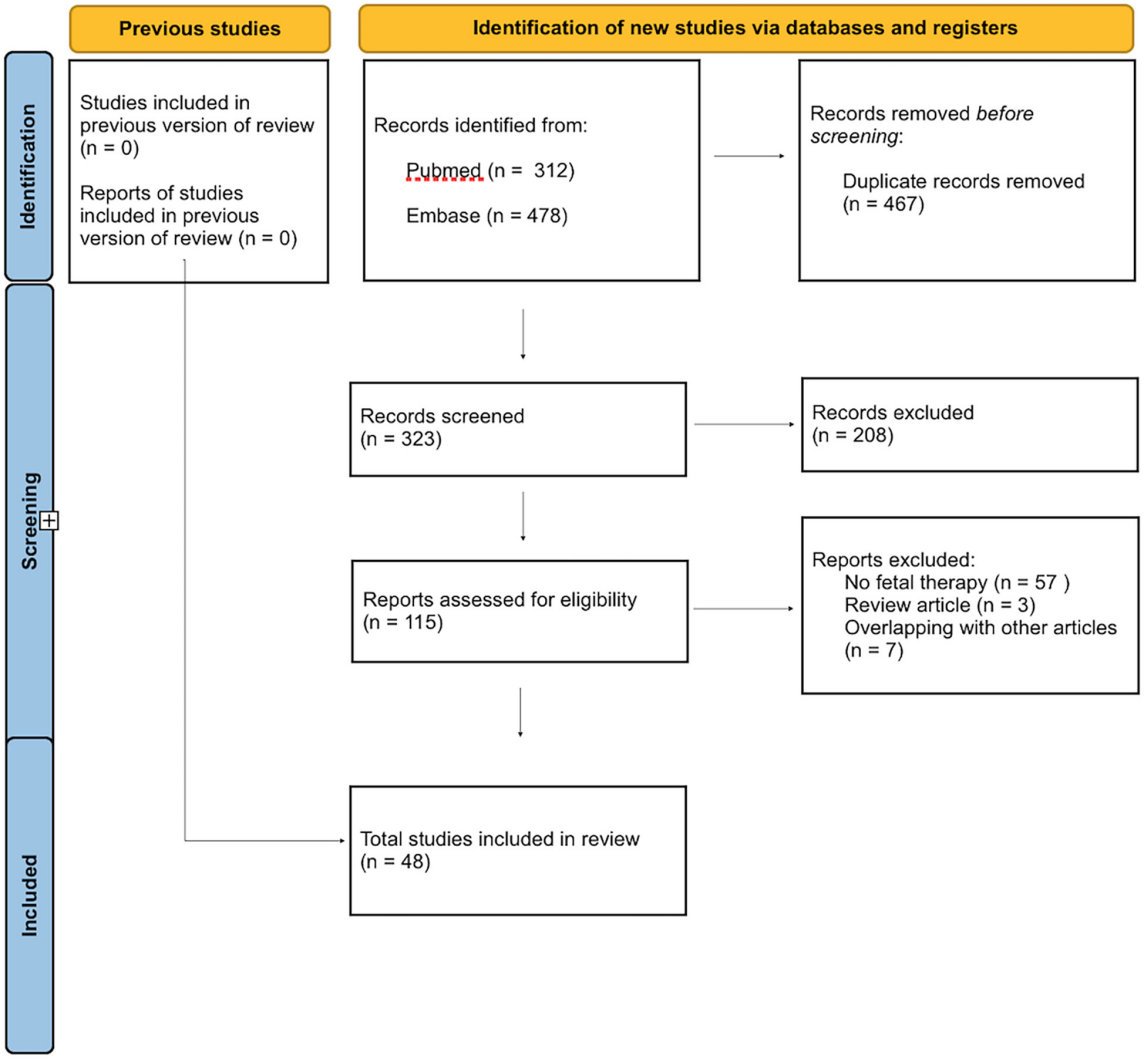
PRISMA flowchart.

A PRISMA flow chart is detailed in Figure 1.

Given the lack of large series, data heterogeneity and the amount of missing data, the results will be exposed in the form of a narrative review.

From the analysis of the 48 included papers, data from 814 cases of FSCT were extracted:out of these, 101 underwent a termination of pregnancy (12,4%).

Overall, *in utero* procedures were attempted on 180 fetuses (22.63%). Mean gestational age (GA) at first fetal procedure was 183 +/- 38 days. Indications were the following: hydrops (30.4%), polyhydramnios (22.1%), cardiac failure (22.1%), risk of preterm labor (6%), mirror syndrome or preeclampsia (3.8%), others (8.8%), unreported (6.7%).

The performed fetal procedures were: amniotic drainage (23.8%), open fetal surgery (21.1%), cyst aspiration (16%), vascular laser ablation (8.8%), interstitial radio-frequency ablation (5.5%), intrauterine transfusion (5%), percutaneous shunting (3.3%), vascular radio- frequency ablation (3.8%), alcohol sclerosis (2.2%), EXIT (2.2%), interstitial laser ablation (1.6%), ascites puncture (1.6%), amnioinfusion (1.1%), histoacryl embolization (0.5%), thermocoagulation by diathermic monopolar (0.5%), unspecified (0.5%).

**Figure 2:**
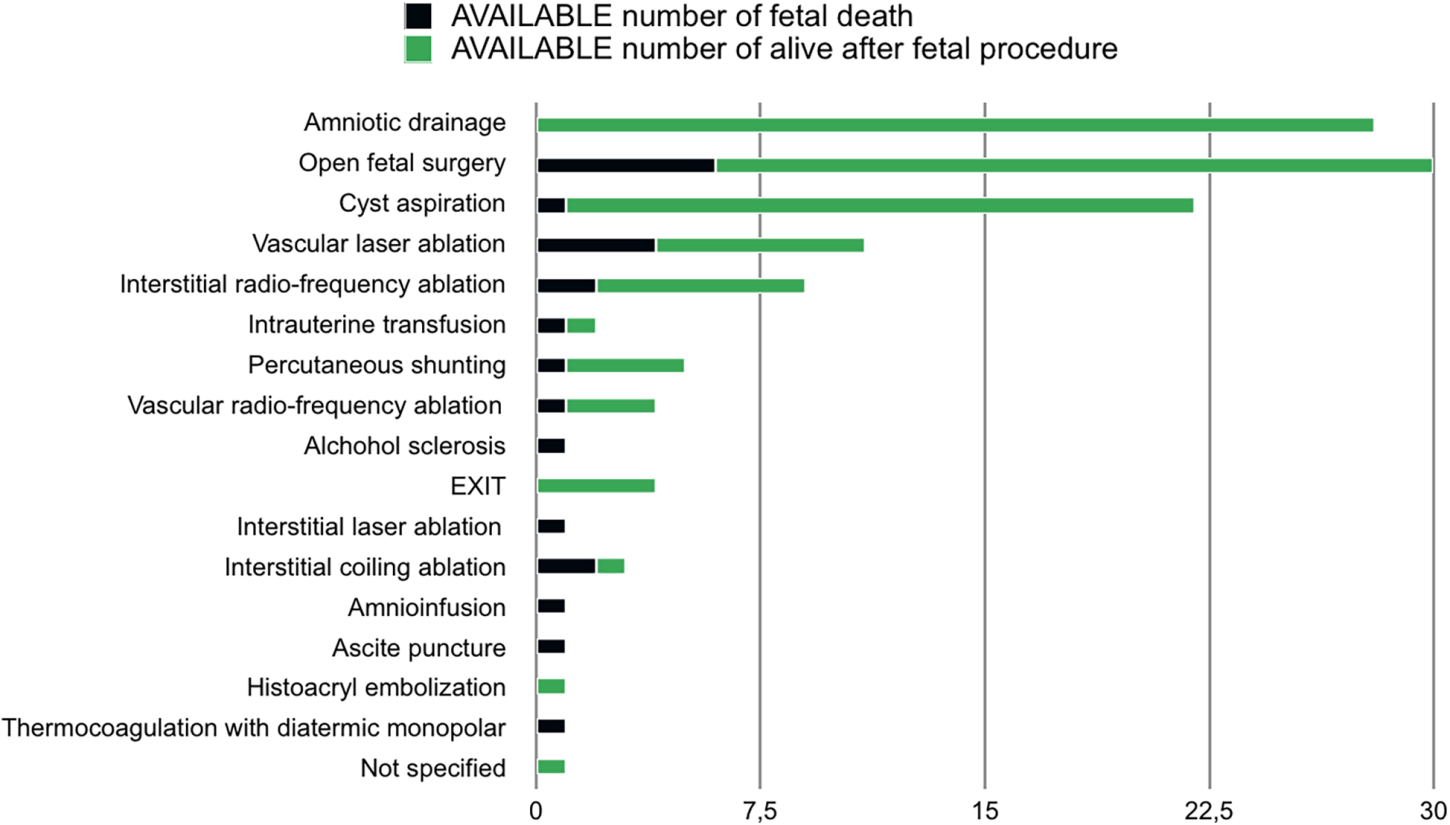
Mortality rate adjusted by number of available data and stratified for type of procedure.

Intrauterine mortality after fetal treatment was 12.8%. Mortality rate stratified for type of procedure is reported in Figure 2.

Time from procedure to death was 8 +/- 6 days (range 0-23): 9 +/- 6 for open surgery, 4 +/- 3 for vascular laser ablation, 21 for interstitial radiofrequency ablation, 2.5 for intrauterine transfusion, 1 for vascular radiofrequency ablation, 0,5 for interstitial laser ablation, 0 for ascite puncture, 23 for amnioinfusion and 2 for thermocoagulation by diathermic monopolar.

**Figure 3:**
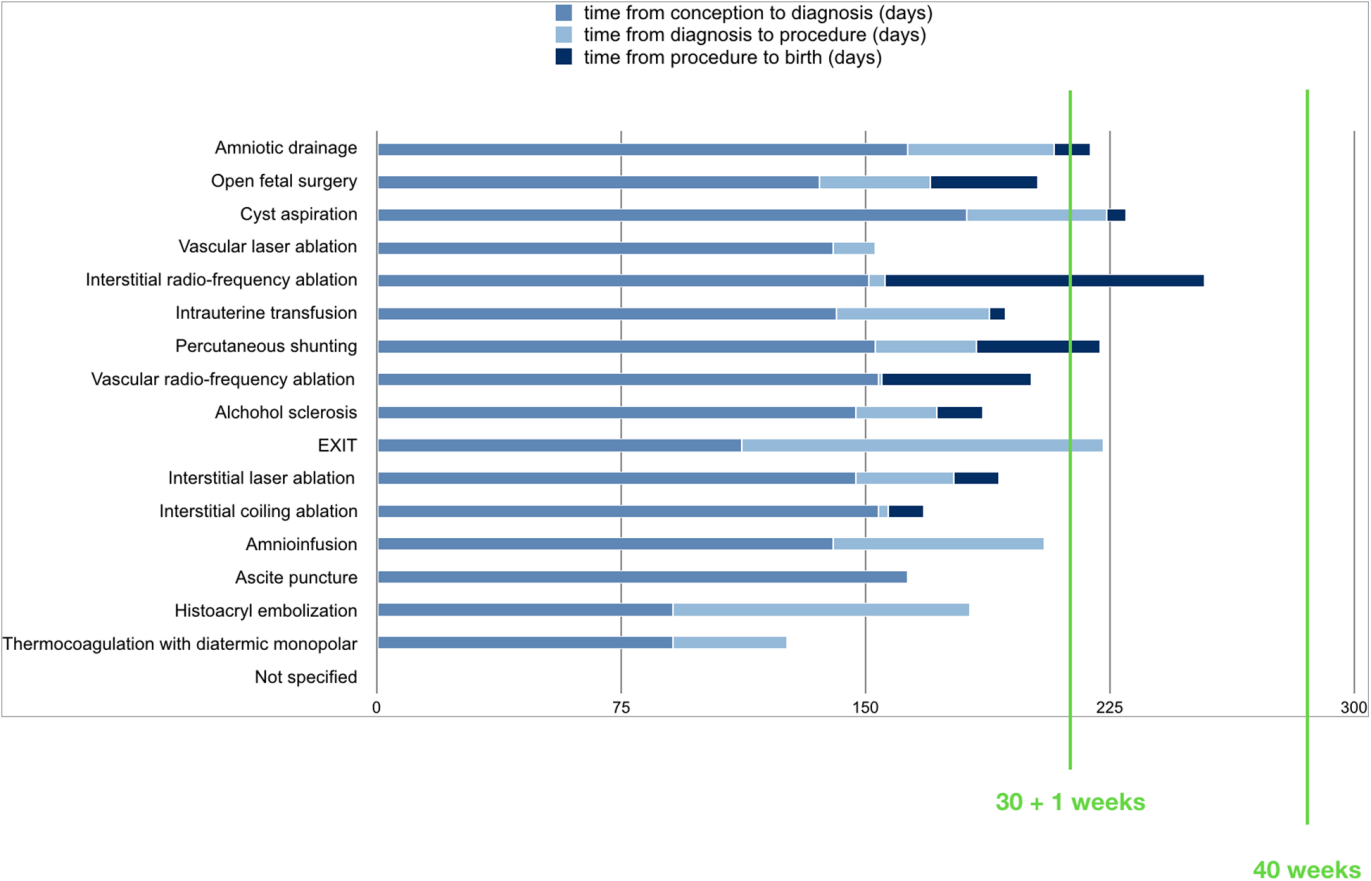
Time from procedure to birth for type of procedure.

Time from diagnosis to treatment was 32 +/- 24 days (range 1–91). Time from procedure to improvement of symptoms was 5,4 +/- 3,2 days (range 3-9). Time from procedure to birth was 27.6 +/- 28 days (range 5–98). Mean gestational age at birth was 207 +/- 24 days (168-254). Time from procedure to birth for type of procedure is reported in Figure 3.

According to available data, overall survival of patients who received a treatment was 57.3% (survival after procedure was not described for 24 patients): 23 died *in utero* due to massive intratumoral hemorrhage (6), persisting or recurrent hydrops (4), cardiac failure (3), tumor rupture (1), unreported (9); 30 died after birth due to complications of prematurity (29) and surgery (1). Maternal complications were HELLPS syndrome (1, maternal death), mirror syndrome (2, resolved after fetal treatment), placental abruption (1).

Despite the small number of cases, a comparison between open fetal surgery and minimally invasive procedures was attempted. Survival rate was 60,9% for open fetal surgery and 58,2% for the remaining procedures. In particular, analysing radiofrequency ablation, laser ablation, alcohol sclerosis, thermocoagulation and histoacryl embolisation survival rate was 44,6%.

## Discussion

Standardized protocols for the management of SCT in neonates define a comprehensive strategy that grants low recurrence rates and optimal survival (17). Inversely, special considerations arise when dealing with FSCT, for which no specific protocols exist and multiple additional variables may play a role in determining an overall dismal prognosis for the fetus and the mother (66, 67). Mass-related complications contribute to creating a challenging and potentially life-threatening condition (2–4) that may, in selected cases, benefit from fetal treatment (5–8).

### Prognostic factors

Despite the small number of cases and the heterogenous procedures, some prognostic factors were identified to assess outcomes in FSCT (10, 64). Tumour volume to fetal weight ratio (TRF) and tumour volume-to-head volume (TV: HV) were recognized as independent predictors of poor fetal outcome and increased maternal operative risk (3, 61). A correlation between solid tumour morphology, placentomegaly, large tumour, high-output cardiac failure/hydrops, polyhydramnios and adverse outcome was also detected (3, 9, 10, 51, 61, 67–69). Including all the previous factors, a prognostic classification for FSCTs was proposed: group A with a tumour diameter of <10 cm, absent or mild vascularity, and slow growth; group B with a diameter of 10 cm or greater, pronounced vascularity or high-output failure and rapid growth; group C with tumour diameter of 10 cm or greater, predominantly cystic lesion with absent or mild vascularity and slow growth. Groups A and C have a good maternal and perinatal outcome, while group B is related to poor outcomes (37, 43).

### Prenatal techniques

Despite some non-negligible limitations, the current review provides an overview of *in utero* approaches for SCT and comprehensively evaluates the full spectrum of currently available modalities (58, 62).

Prenatal management of FSCT is nowadays still debated, but intervention before the development of high-output cardiac failure, hydrops and maternal mirror syndrome are suggested to optimize fetal survival. Overall indications for fetal treatments are therefore based on prognostic factors, clinical features and gestational age (GA). According to this, fetal treatments are suggested for severe FSCT with unsuitable GA for delivery, while FSCT without those complications are candidates for prenatal monitoring and subsequent neonatal surgery. From the analysis of the available literature, the following procedures emerged.

### Ex utero intrapartum treatments (EXIT)

EXIT may be performed in the absence of maternal contraindications, in case of fetuses with an appropriate gestational age (after 32 weeks) and complications such as heart failure and large vascular type I or II tumor necessitating early delivery.Under general anesthesia, to improve uterine relaxation and uteroplacental blood flow, hysterotomy is performed and tumor debulking is carried out under fetal monitoring to interrupt the vascular steal and minimize tumor manipulation and trauma. The fetus is then intubated and given surfactant before the umbilical cord is clamped. EXIT benefits include avoiding intrauterine infection, placental abruption, very early premature rupture of membranes and premature delivery (66). From literature review emerged that good prognosis is achieved, but this technique is not free from risks such as maternal blood loss, infections and increased postpartum recovery time (66). Comfortingly, maternal complications and adverse outcomes are being reduced with the improvement of surgical instruments and techniques as uterine staplers and intraoperative uterine packing.

### Open fetal surgery

In-utero open fetal surgery is otherwise suggested to improve outcomes of selected fetuses with clear miserable prognosis and inappropriate GA for delivery. The following features should be present: impending high-output cardiac failure and related complication as hydrops, fetal GA of 20–30 weeks, isolated type I and II SCT, no associated anomalies and normal fetal karyotype, no maternal contraindication to surgery (7, 8, 23, 30, 36, 43, 64). The procedure aims to debulk the tumor, with formal oncologic resection postponed postnatally. Under general anaesthesia with volatile anaesthetic and intravenous nitroglycerin for uterine relaxation, an anterior hysterotomy is performed away from the margin of the placenta through a Pfannenstiel incision and the fetus is positioned to expose the tumor. The amniotic space is then perfused continuously with warm lactated Ringer's solution, paralytic agent and fentanyl are administered to the fetus via an intramuscular injection. Fetal intravenous access is obtained and continuous echocardiographic and pulse-oximetry monitoring of the fetus is assessed. After FSCT exposition, a tourniquet is applied to constrict blood flow and tumor debulking is performed without attempting to dissect the intrapelvic component or to remove the coccyx. Eventual massive blood loss and coagulopathy should be supervised. Advanced high-output cardiac failure, maternal contraindications to surgery or mirror syndrome, type III-IV SCT and placentomegaly are commonly believed contraindications to fetal surgery. Although open fetal surgery is reported to improve outcomes in such selected fetuses with an overall survival rate of 60,9%, some papers described significant maternal and fetal morbidity as premature rupture of membrane, preeclampsia, gestational diabetes, HELLP syndrome and polyhydramnios/oligohydramnios (29). Intraoperative complications including bleeding, hypovolemia, cardiac arrest, acidosis and coagulation abnormalities are numerous as well. Also postnatal comorbidities ad rectal stenosis, functional constipation, tumor recurrence and wound dehiscence were reported.

Therefore, open fetal surgery and EXITS are down trending procedures and the modified caesarean section (C-section) with immediate tumor resection is nowadays a preferable approach (53).

### Minimally-invasive approaches

Minimally-invasive approaches were proposed to fetuses with severe FSCT and inappropriate GA for delivery who would otherwise have a poor prognosis. The aim is to reduce morbidities related to open fetal procedures and to preserve the fetus in its natural environment without affecting uteroplacental circulation (36, 43, 59–61).

From the analysis of the literature, cyst aspiration and amniotic drainage were the most frequently performed procedures. They are recommended to decrease intrauterine volume and to prevent preterm delivery and tumour rupture if polyhydramnios, hydropic condition or huge volume of the cystic component of the tumour is achieved but gestational age is not appropriate for labour (36, 38, 43). A mean GA at procedure of 29 + 5 weeks for amniotic drainage and 32 weeks for cyst aspiration with a survival rate of 91,4% and 95,4% have been respectively calculated.

Radiofrequency ablation (RFA) is performed in case of rapidly growing, highly vascular mass and cardiomegaly. A transcutaneous introduction of a needle electrode into the tumour under ultrasound (US) guidance is performed and radiofrequency energy (10–100 W) is then delivered causing tissue necrosis. This procedure may involve the entire tumour (“interstitial” ablation) or may be targeted on SCT supply vessels (“vascular” ablation). Results showed that vascular approach aiming to target the feeding vessel reverses the high-output heart failure preventing fetal tumor growth and increasing the survival rate to 64% with resolution of fetal hydrops (7, 32, 46, 59).

On the other hand, interstitial ablation leads to a massive disruption of tumour tissue with higher risk of fetal death due to haemorrhage and activation of disseminated intravascular coagulation with subsequent uncertain prognosis (4, 51). Other risks of the procedure include gas embolization due to microbubbles, hyperkalemia caused by tissue necrosis, perineal damage, hemorrhage, preterm labor and fetal demise. Although RFA has been used as salvage therapy in fetuses who would have otherwise died, the procedure is not free from complications.

Laser ablation is an alternative approach when early delivery is contraindicated because of prematurity and hydrops or cardiac failure affect the fetus (22, 66). Under US guidance a needle of a continuous diode laser is placed into the fetal tumour towards the feeding vessel, without affecting the placenta and, when the tip is in the proper position, laser fibre is passed through the lumen of the needle. Ablation of the abnormal artery is performed in about 3 min. Although some good results were obtained, fetal death after the procedure, related to anaemia and heart failure, were experienced (42). From available literature emerges that laser ablation reduces maternal complications and the risk of premature rupture of membrane, however, the risk of intratumoral necrosis and hemorrhage causing fetal heart failure and thromboembolism increase. Recurrence of hypervascularization, brain injury and early preterm birth (<32 weeks’ gestation) are also described as possible complications (42). Few authors proposed YAG laser ablation of FSCT under fetoscopic view but different outcomes were described (22, 38), speculating that Doppler US guidance seems a safer approach compared to fetoscopic technique.

As an alternative, US guided thermocoagulation of tumour vessels with a diathermic monopolar probe and alcohol sclerosis of tumour's feeding vessels were proposed. Unfortunately, in most cases fetal demise because of persistent cardiac failure was described (33). As a matter of fact, the analysis we performed showed that no fetus survived after thermocoagulation and only 20% were alive after alcohol sclerosis.

As described above, minimally invasive approaches are not free from morbidities. Higher risk of preterm rupture of membranes, early preterm birth < 32 weeks’ gestation and in-utero death, fetal trauma related to thermal spread, extratumoral spread of embolizing or sclerosing agents induced by thermal tissue ablation, haemorrhage and hyperkalemia occur (22, 32, 37, 38, 42, 55, 63).

A recent review speculates that endoscopic laser ablation of vessels on the surface of the tumour and US guided intratumoral injection of alcohol sclerosant have now been abandoned, and the most used techniques are US-guided intratumoral ablation, either by laser or radio frequency (63). However, the few numbers of fetuses prenatally treated is too small to allow conclusions about which of the interventions is the most beneficial.

### Data analysis

Overall, the analysis of the included patients provides crucial insights into prenatally treated SCT: among the 814 included cases, only 180 underwent attempts to fetal therapy, emphasizing the complexity of the decision-making process and the approach. A multidisciplinary evaluation is mandatory.

The primary goal of fetal therapy is to improve fetal survival, halting vascular shunting through the tumor to prevent progression to hydrops, rapid tumor growth and fetal cardiac failure (11, 12, 69): the advances in various techniques indicate a continuous effort to refine and innovate (62, 70). The overall calculated mortality rate following fetal treatment (12.8%) emphasizes the intermixed effects of the severity of the fetal condition and the potential complications or ineffectiveness of the procedure. These results boost the unsolved debate on the balance between delayed treatment and procedure-induced preterm labor. Unfortunately, the correct timing is still unclear: delaying the procedure may result in treating “too late to be effective” while the current life-threatening indications could, by themselves, cause death despite a successful procedure. Unfortunately, due to the lack of comparable data, it is impossible to draw a conclusion.

However, despite the variability among techniques used, the calculated survival rate for fetuses that have access to prenatal treatment is 57%, suggesting substantial benefits of fetal interventions (62, 69, 70), in a subset of patients that would probably have died if left untreated. Hence, reconsidering indication criteria for prenatal treatment and a better knowledge of prenatal treatments complications could offer an opportunity to survive to a larger population of fetuses with FSCT and reduce the pregnancy termination rate.

Concerning a comparison between procedures, nowadays in literature statistically significant data about which approach is more feasible between open surgery and minimally-invasive technique are not available. On the other hand, the analysis performed showed a similar survival rate between open fetal surgery and minimally-invasive techniques (respectively 60,9% and 58,2%) and RFA, laser ablation and amniotic drainage/cyst aspiration seem to be the most effective.

However, given the small number of cases managed with the different treatment modalities, it is difficult to prove any statistically significant superiority between them. In addition, available literature shows that the treatment choice often relies on the team's skills/experience and the facility resources (71, 72), rather than on patient/tumor variables.

As in other rare fetal tumors, networking between centers may give the opportunity to collect data from larger series. This in turn could allow a more meaningful analysis and stronger evidence towards the definition of the best treatment for severe FSCT.

Although no uniform indications have yet been established, the authors of the current paper agreed about an algorithm for FSCT management. In case of low-risk SCTs, prenatal monitoring without intervention and an elective caesarean delivery is warranted. In high-risk SCTs fetuses beyond 28 weeks of gestation with signs of fetal or maternal compromise, early delivery is advised with C-section and subsequent neonatal tumour resection. Since fetal surgery related complications increase after 28 weeks GA, affecting fetus outcome, pre-emptive delivery is recommended to interrupt progression to high-output cardiac failure and subsequent irreversible poor outcome. It would be preferable to deliver a more premature infant in a less compromised state (prehydropic) than to deliver a full term infant in cardiovascular collapse. On the other hand, in type I or II SCT fetuses that develop early signs of hydrops before 28 weeks of gestation prenatal procedures to interrupt the tumour's blood supply may be attempted (32, 47, 50, 60, 66).

In the present review, we did not focus on the impact of fetal treatment on long term follow-up of neonates with FSCT because only few papers properly described their results and correlation with techniques, therefore a comparative analysis between the procedures is not feasible. In fact, in these patients there is a growing concern for long-time functional outcomes related to scars, neurogenic voiding, defecation dysfunction, and associated malformations (62, 64, 69, 70). The potential impact of *in utero* treatments on functional outcomes needs to be part of the debate.

A comparison between outcomes of fetuses with SCT prenatally treated and those who did not receive fetal intervention was not the aim of the current paper. As a matter of fact, fetal treatments are indicated in extreme cases for severe forms of SCT with hydrops or fetal cardiac dysfunction and otherwise poor prognosis, while prenatal expectant management was performed in fetuses with small SCT without significant vascularity and unlikely to develop those complications. In addition, literature lacks data about prenatally detected SCT not underwent prenatal treatment, therefore a comparison of indications and outcomes is nowadays challenging.

## Conclusions

Advances in fetal medicine for SCT offer opportunities to improve outcomes in a group of patients with dismal prognosis. A comprehensive, multidisciplinary evaluation and counseling are crucial in the decision-making process, given the inherent and severe complications associated with fetal treatments.

Unfortunately, the limited sample size and the lack of comparable data highlight the need for further, systematic research as well as multicentric studies and networking to better establish the role of fetal procedures and define the most feasible and safe interventions.

However, despite the variability among techniques used, data suggested benefits of fetal interventions in a subset of patients that would probably have died if left untreated.

## Supplementary material

Supplementary Table 1 Summary of the 48 manuscripts retained for the final analysis.

## References

[1] Gross SJ Benzie RJ Sermer M Skidmore MB Wilson SR. Sacrococcygeal teratoma: prenatal diagnosis and management. Am J Obstet Gynecol. (1987) 156(2):393–6. 10.1016/0002-9378(87)90290-0 3548370

[2] el-Qarmalawi MA Saddik M el Abdel Hadi F Muwaffi R Nageeb K. Diagnosis and management of fetal sacrococcygeal teratoma. Int J Gynaecol Obstet. (1990) 31(3):275–81. 10.1016/0020-7292(90)91023-j 1969370

[3] Akinkuotu AC Coleman A Shue E Sheikh F Hirose S Lim FY Predictors of poor prognosis in prenatally diagnosed sacrococcygeal teratoma: a multiinstitutional review. J Pediatr Surg. (2015) 50(5):771–4. 10.1016/j.jpedsurg.2015.02.034. 25783370

[4] Lee SM Suh DH Kim SY Kim MK Oh S Song SH Antenatal prediction of neonatal survival in sacrococcygeal teratoma. J Ultrasound Med. (2018) 37(8):2003–9. 10.1002/jum.14553 29399854

[5] Bargy F Sapin E. La chirurgie foetale: pour quoi faire? [fetal surgery: for what purpose?]. Pediatrie. (1992) 47(5):347–50. 1331949

[6] Bond SJ. Fetal surgery: correction of anatomic and constitutional defects. J Ky Med Assoc. (1992) 90(5):242–9. 1613337

[7] Wenstrom KD Carr SR. Fetal surgery: principles, indications, and evidence. Obstet Gynecol. (2014) 124(4):817–35. 10.1097/AOG.0000000000000476 25198256

[8] Nelson O Simpao AF Tran KM Lin EE. Fetal anesthesia: intrauterine therapies and immediate postnatalanesthesia for noncardiac surgical interventions. Curr Opin Anaesthesiol. (2020) 33(3):368–73. 10.1097/ACO.0000000000000862 32324666

[9] Okada T Sasaki F Cho K Honda S Naito S Hirokata G Management and outcome in prenatally diagnosed sacrococcygeal teratomas. Pediatr Int. (2008) 50(4):576–80. 10.1111/j.1442-200X.2008.02703.x 18937757

[10] Zhou WX Chen L Zhang YH Wen H. Prenatal diagnosis and prognostic factors analysis of fetal sacrococcygeal teratoma. Zhonghua Fu Chan Ke Za Zhi. (2022) 57(6):413–8. 10.3760/cma.j.cn112141-20220115-00025 35775248

[11] Bond SJ Harrison MR Schmidt KG Silverman NH Flake AW Slotnick RN Death due to high-output cardiac failure in fetal sacrococcygeal teratoma. J Pediatr Surg. (1990) 25(12):1287–91. 10.1016/0022-3468(90)90535-h 2286911

[12] Iacovella C Chandrasekaran N Khalil A Bhide A Papageorghiou A Thilaganathan B. Fetal and placental vascular tumors: persistent fetal hyperdynamic status predisposes to poorer long-term neurodevelopmental outcome. Ultrasound Obstet Gynecol. (2014) 43(6):658–61. 10.1002/uog.13272 24307134

[13] Atis A Kaya B Acar D Polat I Gezdirici A Gedikbasi A. A huge fetal sacrococcygeal teratoma with a vascular disruption sequence. Fetal Pediatr Pathol. (2015) 34(4):212–5. 10.3109/15513815.2015.1042603 26029981

[14] Ibele A Flake A Shaaban A. Survival of a profoundly hydropic fetus with a sacrococcygeal teratoma delivered at 27 weeks of gestation for maternal mirror syndrome. J Pediatr Surg. (2008) 43(8):e17–20. 10.1016/j.jpedsurg.2008.04.010 18675620

[15] Hirose S Farmer DL. Fetal surgery for sacrococcygeal teratoma. Clin Perinatol. (2003) 30(3):493–506. 10.1016/s0095-5108(03)00059-9 14533891

[16] Maselli KM Badillo A. Advances in fetal surgery. Ann Transl Med. (2016) 4(20):394. 10.21037/atm.2016.10.34 27867946 PMC5107396

[17] den Otter SC de Mol AC Eggink AJ van Heijst AF de Bruijn D Wijnen RM. Major sacrococcygeal teratoma in an extreme premature infant: a multidisciplinary approach. Fetal Diagn Ther. (2008) 23(1):41–5. 10.1159/000109225 17934297

[18] Mintz MC Mennuti M Fishman M. Prenatal aspiration of sacrococcygeal teratoma. AJR Am J Roentgenol. (1983) 141(2):367–8. 10.2214/ajr.141.2.367 6603135

[19] Holzgreve W Miny P Anderson R Golbus MS. Experience with 8 cases of prenatally diagnosed sacrococcygeal teratomas. Fetal Ther. (1987) 2(2):88–94. 10.1159/000263289 3332743

[20] Langer JC Harrison MR Schmidt KG Silverman NH Anderson RL Goldberg JD Fetal hydrops and death from sacrococcygeal teratoma:rationale for fetal surgery. Am J Obstet Gynecol. (1989) 160(5 Pt 1):1145–50. 10.1016/0002-9378(89)90177-4 2658603

[21] Bullard KM Harrison MR. Before the horse is out of the barn: fetal surgery for hydrops. Semin Perinatol. (1995) 19(6):462–73. 10.1016/s0146-0005(05)80053-9 8822330

[22] Hecher K Hackelöer BJ. Intrauterine endoscopic laser surgery for fetal sacrococcygeal teratoma. Lancet. (1996) 347(8999):470. 10.1016/s0140-6736(96)90045-8 8618503

[23] Adzick NS Crombleholme TM Morgan MA Quinn TM. A rapidly growing fetal teratoma. Lancet. (1997) 349(9051):538. 10.1016/S0140-6736(97)80088-8 9048793

[24] Chisholm CA Heider AL Kuller JA von Allmen D McMahon MJ Chescheir NC. Prenatal diagnosis and perinatal management of fetal sacrococcygeal teratoma. Am J Perinatol. (1998) 15(8):503–5. 10.1055/s-2007-994074 9788651

[25] Garcia AM Morgan WM 3rd Bruner JP. In utero decompression of a cystic grade IV sacrococcygeal teratoma. Fetal Diagn Ther. (1998) 13(5):305–8. 10.1159/000020859 9813425

[26] Kay S Khalife S Laberge JM Shaw K Morin L Flageole H. Prenatal percutaneous needle drainage of cystic sacrococcygeal teratomas. J Pediatr Surg. (1999) 34(7):1148–51. 10.1016/s0022-3468(99)90587-0 10442611

[27] Kitano Y Flake AW Crombleholme TM Johnson MP Adzick NS. Open fetal surgery for life-threatening fetal malformations. Semin Perinatol. (1999) 23(6):448–61. 10.1016/s0146-0005(99)80024-x 10630541

[28] Chiba T Albanese CT Jennings RW Filly RA Farrell JA Harrison MR. In utero repair of rectal atresia after complete resection of a sacrococcygeal teratoma. Fetal Diagn Ther. (2000) 15(3):187–90. 10.1159/000021003 10782007

[29] Goto M Makino Y Tamura R Ikeda S Kawarabayashi T. Sacrococcygeal teratoma with hydrops fetalisand bilateral hydronephrosis. J Perinat Med. (2000) 28(5):414–8. 10.1515/JPM.2000.054 11125934

[30] Graf JL Albanese CT Jennings RW Farrell JA Harrison MR. Successful fetal sacrococcygeal teratoma resection in a hydropic fetus. J Pediatr Surg. (2000) 35(10):1489–91. 10.1053/jpsu.2000.16420 11051157

[31] Jouannic JM Dommergues M Auber F Bessis R Nihoul-Fekete C Dumez Y. Successful intrauterine shunting of a sacrococcygeal teratoma (SCT) causing fetal bladder obstruction. Prenat Diagn. (2001) 21(10):824–6. 10.1002/pd.147 11746122

[32] Paek BW Jennings RW Harrison MR Filly RA Tacy TA Farmer DL Radiofrequency ablation of human fetal sacrococcygeal teratoma. Am J Obstet Gynecol. (2001) 184(3):503–7. 10.1067/mob.2001.110446 11228510

[33] Lam YH Tang MH Shek TW. Thermocoagulation of fetal sacrococcygeal teratoma. Prenat Diagn. (2002) 22(2):99–101. 10.1002/pd.246 11857611

[34] Ibrahim D Ho E Scherl SA Sullivan CM. Newborn with an open posterior hip dislocation and sciatic nerve injury after intrauterine radiofrequency ablation of a sacrococcygeal teratoma. J Pediatr Surg. (2003) 38(2):248–50. 10.1053/jpsu.2003.50055 12596115

[35] Wilson RD Johnson MP Crombleholme TM Flake AW Hedrick HL King M Chorioamniotic membrane separation following open fetal surgery: pregnancy outcome. Fetal Diagn Ther. (2003) 18(5):314–20. 10.1159/000071972 12913340

[36] Hedrick HL Flake AW Crombleholme TM Howell LJ Johnson MP Wilson RD Sacrococcygeal teratoma: prenatal assessment, fetal intervention, and outcome. J Pediatr Surg. (2004) 39(3):430–8. 10.1016/j.jpedsurg.2003.11.005 15017565

[37] Benachi A Durin L Vasseur Maurer S Aubry MC Parat S Herlicoviez M Prenatally diagnosed sacrococcygeal teratoma: a prognostic classification. J Pediatr Surg. (2006) 41(9):1517–21. 10.1016/j.jpedsurg.2006.05.009 16952584

[38] Makin EC Hyett J Ade-Ajayi N Patel S Nicolaides K Davenport M. Outcome of antenatally diagnosed sacrococcygeal teratomas: single-center experience (1993–2004). J Pediatr Surg. (2006) 41(2):388–93. 10.1016/j.jpedsurg.2005.11.017 16481257

[39] Perrotin F Herbeteau D Machet MC Potin J Lardy H Arbeille P. In utero doppler ultrasound-guided embolization for the treatment of a large, vascular sacrococcygeal teratoma causing fetal hydrops OP06.20. Ultrasound Obstet Gynecol. (2006) 28(4):458–9. 10.1002/uog.3198

[40] Friédérich L Diguet A Eurin D Bachy B Roman H Marpeau L Tératome sacrococcygiende la taille du faetus: surveillance anténatale, thérapeutique faetale in utero et prise en charge obstétricale [A voluminous sacrococcygeal teratoma: prenatal diagnosis, in-utero treatment and obstetric management]. Gynecol Obstet Fertil. (2007) 35(10):1001–4. 10.1016/j.gyobfe.2007.07.030 17921039

[41] Adzick NS. Open fetal surgery for life-threatening fetal anomalies. Semin Fetal Neonatal Med. (2010) 15(1):1–8. 10.1016/j.siny.2009.05.003 19540178

[42] Ruano R Duarte S Zugaib M. Percutaneous laser ablation of sacrococcygeal teratoma in a hydropic fetus with severe heart failure–too late for a surgical procedure? Fetal Diagn Ther. (2009) 25(1):26–30. 10.1159/000188663 19129708

[43] Wilson RD Hedrick H Flake AW Johnson MP Bebbington MW Mann S Sacrococcygeal teratomas: prenatal surveillance, growth and pregnancy outcome. Fetal Diagn Ther. (2009) 25(1):15–20. 10.1159/000188056 19122459

[44] Zhang ZT Liu CX Zhou YZ Li QL Wang WL Huang Y Intrapartum operation on fetuses with birth defects and its outcome. Zhonghua Fu Chan Ke Za Zhi. (2010) 45(9):652–7. 10.3760/cma.j.issn.0529-567x.2010.09.004 21092543

[45] Amann C Geipel A Müller A Heep A Ritgen J Stressig R Fetal anemia of unknown cause–a diagnostic challenge. Ultraschall Med. (2011) 32(Suppl 2):E134–40. 10.1055/s-0031-1281756 22161617

[46] Lee MY Won HS Hyun MK Lee HY Shim JY Lee PR Perinatal outcome of sacrococcygeal teratoma. Prenat Diagn. (2011) 31(13):1217–21. 10.1002/pd.2865 22024911

[47] Roybal JL Moldenhauer JS Khalek N Bebbington MW Johnson MP Hedrick HL Early delivery as an alternative management strategy for selected high-risk fetal sacrococcygeal teratomas. J Pediatr Surg. (2011) 46(7):1325–32. 10.1016/j.jpedsurg.2010.10.020 21763829

[48] Stefanovic V Halmesmäki E. Peripartum ultrasound-guided drainage of cystic fetal sacrococcygeal teratoma for the prevention of the labor dystocia: a report of two cases. AJP Rep. (2011) 1(2):87–90. 10.1055/s-0031-1284220 23705093 PMC3653534

[49] Wee WW Tagore S Tan JV Yeo GS. Foetal sacrococcygeal teratoma: extremes in clinical presentation. Singapore Med J. (2011) 52(6):e118–23. 21731981

[50] Cass DL Olutoye OO Ayres NA Moise KJ Jr Altman CA Johnson A Defining hydrops and indications for open fetal surgery for fetuses with lung masses and vascular tumors. J Pediatr Surg. (2012) 47(1):40–5. 10.1016/j.jpedsurg.2011.10.019 22244390

[51] Usui N Kitano Y Sago H Kanamori Y Yoneda A Nakamura T Outcomesof prenatally diagnosed sacrococcygeal teratomas: the results of a Japanese nationwide survey. J Pediatr Surg. (2012) 47(3):441–7. 10.1016/j.jpedsurg.2011.08.020 22424335

[52] Goto S Suzumori N Obayashi S Ozaki Y Sugiura-Ogasawara M. Two cases of prenatally diagnosed sacrococcygeal teratoma type I with different clinical features. Congenit Anom (Kyoto). (2013) 53(2):92–4. 10.1111/j.1741-4520.2012.00369.x 23751044

[53] Van Mieghem T Al-Ibrahim A Deprest J Lewi L Langer JC Baud D Minimally invasive therapy for fetal sacrococcygeal teratoma: case series and systematic review of the literature. Ultrasound Obstet Gynecol. (2014) 43(6):611–9. 10.1002/uog.13315 24488859

[54] Ayed A Tonks AM Lander A Kilby MD. A review of pregnancies complicated by congenital sacrococcygeal teratoma in the west midlands region over an 18-year period: population-based, cohortstudy. Prenat Diagn. (2015) 35(11):1037–47. 10.1002/pd.4641 26114890

[55] Arisoy R Erdogdu E Kumru P Demirci O Ergin N Pekin O Prenatal diagnosis and outcomes of fetal teratomas. J Clin Ultrasound. (2016) 44(2):118–25. 10.1002/jcu.22310 26426797

[56] Peiró JL Sbragia L Scorletti F Lim FY Shaaban A. Management of fetal teratomas. Pediatr Surg Int. (2016) 32(7):635–47. 10.1007/s00383-016-3892-3 27112491

[57] Sananes N Javadian P Schwach Werneck Britto I Meyer N Koch A Gaudineau A Technical aspects and effectiveness of percutaneous fetal therapies for large sacrococcygeal teratomas: cohort study and literature review. Ultrasound Obstet Gynecol. (2016) 47(6):712–9. 10.1002/uog.14935 26138446

[58] Baumgarten HD Gebb JS Khalek N Moldenhauer JS Johnson MP Peranteau WH Preemptive delivery and immediate resection for fetuses with high-risk sacrococcygeal teratomas. Fetal Diagn Ther. (2019) 45(3):137–44. 10.1159/000487542 29734172

[59] Gebb JS Khalek N Qamar H Johnson MP Oliver ER Coleman BG High tumor volume to fetal weight ratio is associated with worse fetal outcomes and increased maternal risk in fetuses with sacrococcygeal teratoma. Fetal Diagn Ther. (2019) 45(2):94–101. 10.1159/000486782 29495013

[60] Wohlmuth C Bergh E Bell C Johnson A Moise KJ Jr van Gemert MJC Clinical monitoring of sacrococcygeal teratoma. Fetal Diagn Ther. (2019) 46(5):333–40. 10.1159/000496841 30893693

[61] Litwińska M Litwińska E Janiak K Piaseczna-Piotrowska A Szaflik K. Percutaneous intratumor Laser ablation for fetal sacrococcygeal teratoma. Fetal Diagn Ther. (2020) 47(2):138–44. 10.1159/000500775 31291630

[62] Cass DL. Fetal abdominal tumors and cysts. Transl Pediatr. (2021) 10(5):1530–41. 10.21037/tp-20-440 34189111 PMC8192983

[63] Simonini C Strizek B Berg C Gembruch U Mueller A Heydweiller A Fetal teratomas - A retrospective observational single-center study. Prenat Diagn. (2021) 41(3):301–7. 10.1002/pd.5872 33242216

[64] Van Heurn LJ Coumans ABC Derikx JPM Bekker MN Bilardo KM Duin LK Factors associated with poor outcome in fetuses prenatally diagnosed with sacrococcygeal teratoma. Prenat Diagn. (2021) 41(11):1430–8. 10.1002/pd.6026 34327722 PMC9292788

[65] Ding Y Yang M Lv M Jiang Y Dong T Zhao B The ex-utero intrapartum treatment (EXIT) strategy for fetal giant sacrococcygeal teratoma with cardiac insufficiency: a case report and review of the literature. Front Oncol. (2022) 12:1035058. 10.3389/fonc.2022.1035058 36408142 PMC9666771

[66] Coleman A Shaaban A Keswani S Lim FY. Sacrococcygeal teratoma growth rate predicts adverse outcomes. J Pediatr Surg. (2014) 49(6):985–9. 10.1016/j.jpedsurg.2014.01.036 24888848

[67] Zheng XQ Yan JY Xu RL Wang XC Chen X Huang KH. A clinical analysis of the diagnosis and treatment of fetal sacrococcygeal teratomas. Cancer Manag Res. (2020) 12:13185–93. 10.2147/CMAR.S287682 33380826 PMC7767721

[68] Coleman A Kline-Fath B Keswani S Lim FY. Prenatal solid tumor volume index: novel prenatal predictor of adverse outcome in sacrococcygeal teratoma. J Surg Res. (2013) 184(1):330–6. 10.1016/j.jss.2013.05.029 23773720

[69] Özsürmeli M Büyükkurt S Sucu M Arslan E Mısırlıoğlu S Akçabay Ç Evaluation of prenatally diagnosed fetal sacrococcygeal teratomas: a case series of seventeen pregnancies from south-central Turkey. Turk J Obstet Gynecol. (2020) 17(3):170–4. 10.4274/tjod.galenos.2020.68812 33072420 PMC7538821

[70] Kum CK Wong YC Prabhakaran K. Management of fetal sacroccocygeal teratoma. Ann Acad Med Singap. (1993) 22(3):377–80. 8373123

[71] Sy ED Lee H Ball R Farrell J Poder L Nobuhara KK Spontaneous rupture of fetal sacrococcygeal teratoma. Fetal Diagn Ther. (2006) 21(5):424–7. 10.1159/000093884 16912491

[72] Yamaguchi Y Tsukimori K Hojo S Nakanami N Nozaki M Masumoto K Spontaneous rupture of sacrococcygeal teratoma associated with acute fetal anemia. Ultrasound Obstet Gynecol. (2006) 28(5):720–2. 10.1002/uog.3821 16958151

