# Supplementary material for: Fetal treatment of sacrococcygeal teratoma: state of the art
Source: Front Pediatr. 2025 Jun 10;13:1410269. doi: 10.3389/fped.2025.1410269 (PMC12185440; doi:10.3389/fped.2025.1410269)
Supplement: Supplementary Table 1 — Summary of the 48 manuscripts retained for the final analysis. [file Datasheet1.pdf]

Reference	Tot SCTs (n)	Termination of pregnancy (n)	Fetal treatment (n)	GA at fetal treatment (w)	Type of fetal Intervention	Alive after fetal intervention (n)
**Mintz 1983** [18]	1	0	1	36 + 1	Cyst aspiration (1)	1
**Holzgreve 1987** [19]	8	2	1	31	Amniotic drainage (1)	0
**Langer 1989** [20]	7	0	1	24 + 2	Open fetal surgery (1)	0
**Bullard 1995** [21]	6	0	2	24, 26	Open fetal surgery (2)	0
**Hecher 1996** [22]	1	0	1	20	Vascular laser ablation and transfusion (1)	1
**Adzick 1997** [23]	1	0	1	25	Open fetal surgery (1)	1
**Chisholm 1998** [24]	9	0	3	NA	Amniotic drainage (3)	3
**Garcia 1998** [25]	1	0	3	22, 24, 25	Cyst aspiration (2), percutaneous shunting(1)	1
**Kay 1999** [26]	2	0	2	37 + 5, 39 + 5	Cyst aspiration (2)	2
**Kitano 1999** [27]	1	0	1	26	Open fetal surgery (1)	1
**Chiba 2000** [28]	1	0	1	27	Open fetal surgery (1)	0
**Goto 2000** [29]	1	0	1	28 + 3	Vesico-amniotic shunt (1)	0
**Graf 2000** [30]	1	0	1	23	Open fetal surgery (1)	1
**Jouannic 2001** [31]	1	0	1	28	Percutaneous shunting (1)	1
**Paek 2001** [32]	4	1	4	20, 22	Radiofrequency interstitial ablation (2), radiofrequency vascular ablation (2)	2
**Lam 2002** [33]	1	0	1	18	Thermocoagulation with diathermic monopolar (1)	0
**Ibrahim 2003** [34]	1	0	1	18	Vascular radiofrequency ablation(1)	1
**Wilson 2003** [35]	2	0	2	23	Open fetal surgery (2)	2
**Hedrick 2004** [36]	30	4	14	24-36, 29, 21, 23+6, 25+1, 26	Cyst aspiration (6), amniotic drainage (3), amnioinfusion (1), open fetal surgery (4)	12
**Benachi 2006** [37]	44	4	1	NA	Interstial coiling ablation (1)	0
**Makin 2006** [38]	41	6	12	NA	Vascular laser ablation (4), alcohol sclerosis (3), cyst drainage (2), amniotic drainage (2), vescicoamniotic shunt (1)	6
**Perrotin 2006** [39]	1	0	1	26	Histoacryl embolization (1)	1
**Friederich 2007** [40]	1	0	1	28	Amniotic drainage (1)	1
**Adzick 2009** [41]	70	0	4	22-26	Open fetal surgery (4)	3
**Ruano 2009** [42]	1	0	1	24	Vascular laser ablation (1)	0
**Wilson 2009** [43]	23	4	5	26 + 2, 24 + 4, 36 + 1, 31, 29+2	Amniotic drainage (4), amnioinfusion (1)	3
**Zhang 2010** [44]	1	0	1	NA	EXIT (1)	1
**Amann 2011** [45]	2	0	2	14-34	Intrauterine transfusion (2)	NA
**Lee 2011** [46]	22	NA	8	20-3, 23 + 3	Radiofrequency interstitial ablation (6), tumor-amniotic shunt (1), abdomina-amniotic shunt (1)	6
**Roybal 2011** [47]	35	0	6	26+3, 22-26	EXIT (1), open fetal surgery (5)	5
**Stefanovic 2011** [48]	2	0	2	32, 38	Cyst aspiration (2)	2
**Wee 2011** [49]	2	0	1	24 +5, 26 +3, 27 + 6, 28+6	Intrauterine transfusion (1), intrauterine transfusion and amniotic drainage (1), intrauterine transfusion (1), intrauterine transfusion (1)	0
**Cass 2012** [50]	12	0	1	NA	Open fetal surgery (1)	1
**Usui 2012** [51]	97	11	15	NA	Radiofrequency interstitial ablation (1), amniotic drainage (11), cyst aspiration (2), ascite puncture (1)	11
**Goto 2013** [52]	2	0	1	27	Amniotic drainage (1)	1
**Van Mieghem 2014** [53]	5	0	5	22, 26 + 3, 26 + 6, 17 + 5, 26 + 6	Radiofrequency ablation (2), vascular laser ablation (1), interstitial coiling ablation (2)	2
**Ayed 2015** [54]	23	7	5		Amniotic drainage (5)	5
**Arisoy 2016** [55]	12	6	1	37	EXIT (1)	1
**Peirò 2016** [56]	55	0	7	/	Open fetal surgery (7)	3
**Sananes 2016** [57]	13	0	5	21-22, 24, 23	Vascular laser ablation (3), interstitial laser ablation (1), alcohol sclerosis (1)	2
**Baumgarten 2019** [58]	42	0	4	<26, 28, 32	Open fetal surgery (2), amniotic drainage (2)	3
**Gebb 2019** [59]	58	27	5	22-24, 29	Open fetal surgery(4), cyst aspiration (1)	2
**Wohlmuth 2019** [60]	12	0	1	23+6	Unknown (1)	1
**Litwinska 2020** [61]	7	0	7	20,21,23,**20**,19,19,23	Vascular laser ablation (7)	3
**Cass 2021** [62]	2	0	2	23, 25	Open fetal surgery (2)	2
**Simonini 2021** [63]	47	9	22	25-39	Amniotic drainage (7), radiofrequency ablation (3), intrauterine transfusion (3), cyst aspiration (7), ascite puncture (2)	NA
**Van Heurn 2021** [64]	84	18	11	25, 26 +3, 28, 33+5, 25, 21, 31, 38, 40	Intrauterine trasfusion (4), amniodrainage (2), interstitial laser coagulation (1), cyst aspiration (4)	7
**Ding 2022** [65]	1	0	1	32	EXIT (1)	1
